# Prevalence of multiple long-term chronic conditions and associated disabilities among community-dwelling adults in Riyadh

**DOI:** 10.3389/fpubh.2024.1275124

**Published:** 2024-10-03

**Authors:** Aqeel M. Alenazi, Norah A. Alhwoaimel, Bader A. Alqahtani, Mohammed M. Alshehri, Ahmed S. Alhowimel, Kamlesh Khunti

**Affiliations:** ^1^Department of Health and Rehabilitation Sciences, Prince Sattam Bin Abdulaziz University, Al-Kharj, Saudi Arabia; ^2^Department of Physical Therapy, Jazan University, Jizan, Saudi Arabia; ^3^Diabetes Research Centre, Leicester General Hospital, University of Leicester, Leicester, United Kingdom

**Keywords:** disabilities, impairments, functional limitations, multiple chronic diseases, multimorbidity

## Abstract

**Background/objectives:**

Saudi Arabia is experiencing a rapid increase in chronic diseases and disabilities. However, there is a dearth of research on these topics in the Arab world. This study aimed to examine the prevalence of multiple long-term chronic conditions (MLTCs) and disabilities and their relationship.

**Methods:**

The survey was conducted in Riyadh, Saudi Arabia, in 2023. Convenient sampling was used to select 324 participants aged 50 years and older, using data on disabilities status. The survey collected information on age, sex, body mass index (BMI), MLTCs or multi-morbidity, and activities of daily living (ADL). Disabilities was measured using Arabic versions of basic ADL and the Barthel index.

**Results:**

The prevalence of MLTCs among participants was 49.4%. The prevalence of disabilities measured using the ADL and Barthel index was 33.6 and 49.7%, respectively, and these rates increased by 42.5 and 58.1% among participants with MLTCs (*n* = 160). MLTCs were associated with an increased risk of disabilities using ADL [odds ratio (OR) 1.99, *p* = 0.037] and the Barthel index (OR 2.27, *p* = 0.007).

**Conclusion:**

Approximately half of the participants with disabilities data had MLTCs, and approximately a third to half had a disabilities. MLTCs were significantly associated with various types of disabilities among community-dwelling Saudi adults. Hence, strategies to reduce chronic diseases may result in a reduction in disabilities, and vice versa.

## Introduction

MLTCs, known as multimorbidity, are defined as having two chronic diseases or more (1). Different reports have estimated the prevalence of MLTCs ranging from 16.6% in Ghana to 45.3% in Russia (2). A recent estimated prevalence of MLTCs in community settings was 33.1% using different studies (3). Although this study comprehensively included studies from high-income countries (i.e., Australia, Canada, Spain, Germany, Portugal, the United Kingdom, and others) and low-and middle-income countries (i.e., China, India, Brazil, South Africa, Ghana, Pakistan, Egypt, Iran, and others), other countries such as Saudi Arabia were not included due to a lack of reports. One study examined the independent association between MLTCs and disabilities with the risk of falling without considering MLTCs with disabilities (4). Another study examined the MLTCs among hospitalized patients with COVID-19 in Saudi Arabia (5).

Negative clinical and financial outcomes have been linked with MLTCs (6). The associated cost with chronic diseases was estimated at 75% of the total health expenditure, ranging from hospitalization, medications, consultations, transportation, and rehabilitation services (7, 8). The main goal of healthcare is to maintain quality of life and independent living among persons with MLTCs. Prior evidence has shown that MLTCs were associated with negative health outcomes, including mortality, increased use of health services, polypharmacy, and disabilities (9, 10).

The term “disabilities” is an umbrella term encompassing impairments, activity limitations, and participation limitations experienced by an individual (11). A person is considered disabled when they experience a partial or complete dependence for performing ADL. The prevalence of disabilities has been estimated between 16.2% in China and 55.7% in India (2). In Saudi Arabia, a recent report has estimated the prevalence of disabilities at a national level at 3.3% per 100,000 persons (12). It has been suggested that disabilities is associated with extra cost depending on the severity and life cycle (13, 14). The association between MLTCs and disabilities has been studied previously, revealing a strong relationship (9, 15–21). However, these studies included older adults and were conducted in countries other than Saudi Arabia. It is of interest to examine the prevalence and association of MLTCs and disabilities in Saudi Arabia for several reasons. The prevalence of chronic diseases in the Saudi population has been increasing recently, affecting both older and younger age groups (22–28). Urbanization and inactive lifestyles such as lack of physical activity and sedentary behavior have a negative impact on health in Saudi Arabia (29). Our previous report found that only 17.40% of the Saudi population participated in the recommended physical activity level (i.e., ≥ 150 min per week) (29). Previous studies have mainly focused on older adults, with limited evidence concerning adults aged 50 years and older. This age group has been underrepresented in research, as it falls between the categories of older adults and younger adults. However, this age group is crucial as it often represents a transition period to chronic diseases and declined health. Therefore, it is crucial to explore MLTCs and disabilities in this population to determine etiological factors, establish priorities for healthcare, and develop preventive and therapeutic strategies for these conditions. Hence, the aims of this study were: (1) to explore the prevalence of MLTCs and disabilities among adults aged 50 years and older and (2) to examine the association between MLTCs and disabilities using the basic ADL and Barthel index in this population. We hypothesized that having MLTCs, two chronic diseases or more would be associated with any level of disabilities using the ADL and Barthel index among Saudi community-dwelling individuals aged 50 years and older living in Riyadh. Having any kind of disabilities on each scale was recorded as having a disabilities in the ADL and Barthel index.

## Methods

### Study design and participants

A survey was conducted to ascertain the prevalence of MLTCs or multimorbidity and disabilities, as well as to evaluate the association between MLTCs and disabilities using the basic ADL and Barthel index for ADL scales. Data were collected from community-dwelling older adults who lived in Riyadh, Saudi Arabia. This study included a total of 324 participants aged between 50 and 91 years. The inclusion criteria included the age of 50 years and older, being able to read and write Arabic, having a disabilities status record, and Saudi citizenship. The data collection started in February 2023 to June 2023. Many community locations were used for recruitment, including malls, mosques, clinics, and social gathering places. The data collection was conducted in person by an independently trained physiotherapy researcher, starting with demographic and clinical data, including age, gender, BMI, and chronic diseases. The included participants signed a consent form following the Declaration of Helsinki. This study was approved by the Research Ethics Committee at Prince Sattam Bin Abdulaziz University (No. RHPT/022/010).

### Demographics and clinical variables

Age was recorded in years and categorized into two groups (i.e., < 65 years and ≥ 65 years). BMI was calculated by dividing weight in kg by height in m^2^ and categorized into three groups [i.e., normal weight (BMI < 25), overweight (25 < BMI <30), and obese (BMI ≥ 30)]. Major chronic diseases were collected via self-reported diagnoses, including hypertension, diabetes, cardiovascular disease, lung disease, neurological diseases (i.e., stroke, Parkinson’s disease, and spinal cord injury), cancer, and arthritis.

### Outcome measures

Disabilities was measured using an Arabic version of basic ADL and the Barthel index for ADL scales (30, 31). The Barthel index for the ADL scale was added as the translated Arabic version of the original ADL scale has not been validated, since it used a different scoring system. The Arabic ADL used 0, 0.5, or 1.0 for each item, while the original scale used 0 and 1 score for each item.

The ADL is a 6-item scale that assesses overall functional activity in (1) bathing, (2) dressing, (3) going to the toilet, (4) transferring (movement), (5) continence, and (6) feeding. The Arabic version of ADL has a high validity and reliability level because it has been established in an Arabic older adult population living in nursing homes (30). The six components of the ADL scale are scored 0, 0.5, or 1. The total ADL score ranges from 0 to 6, where 0 indicates very dependent, 1–5 points indicates partially dependent, and 6 indicates complete independence. Another calculation has been performed according to the developer to convert the scores into 100 by adding the scores on the 6 items and dividing by 6. Then, multiply the results by 100 to be converted (30, 32). For the current study, any participant with a score less than 100 was considered to have a disabilities (20, 33, 34). This approach has been used in previous studies from the community (20, 33, 34).

The Barthel index of ADL (1989) was also used to measure ADL in the study population (35). This index includes 10 items related to basic ADL consisting of bowel control, bladder control, grooming, feeding, toilet use, transfer, mobility, dressing, climbing stairs, and bathing. Each item scored between 0 and 15, depending on the condition of the individual and the nature of the item. The total score for each person varies from 0 (maximum disabilities and dependency) to 100 (maximum strength and independence). The Barthel index has been translated into multiple languages. However, no study has translated this scale into Arabic. Therefore, we performed a cross-cultural adaptation and validation for translating the Barthel index using the international guidelines by Beaton criteria following a 5-step process. These steps included forward translation, synthesis of translation, backward translation, expert committee review, and pre-final version testing. The results of this process will be published elsewhere. For the current study, any participant with a score less than 100 was considered to have a disabilities, and this approach has been used in previous studies with a similar sample of community-dwelling older adults (20, 33, 34, 36).

### Sample size calculation

The sample has been calculated based on past evidence related to the prevalence of MLTCs (3). The reported prevalence of MLTCs was 33.1%. Therefore, we calculated the sample size using this formula [*n* = Z^2^ P(1-P)/d^2^], where n = sample size, Z = Z score statistic for a level of confidence (1.96), P = the prevalence of MLTCs (33.1%), and d = the degree of precision (0.05). The sample size was estimated to be 340.

### Statistical analysis

The data were expressed as counts and percentages for categorical variables. Disabilities was dichotomized into yes (if the score was not the maximum independence) or no (if the score was full, indicating maximum independence). The chi-square test was used for comparisons between categorical variables across MLTC classification.

To examine the association between MLTCs and disabilities using ADL and the Barthel index, a generalized linear model with binary logistic regression was used. This model allows for expressing the odds for each classification of the MLTCs (none, one disease, and two diseases or more). Two models were used, one with disabilities (yes, no) using ADL and the second with disabilities (yes, no) using the Barthel index. All models were adjusted for age, sex, and BMI categories. Missing data were handled using the case-wise deletion method. An alpha level of 0.05 was used in the analysis, which was conducted using IBM SPSS for Mac version 25.0 (SPSS Inc., Chicago, IL, United States).

## Results

A total of 324 participants aged 50 years and older with records of disabilities were included in the current study, with a total of 143 (44.1%) participants being older adults (age > 65) and a total of 193 (59.6%) participants being women. The prevalence of MLTCs was 49.4%. The prevalence of disabilities using ADL and the Barthel index was 33.6 and 49.7%, respectively. Table 1 shows the demographics and clinical characteristics of all participants classified based on MLTCs, including none, one chronic disease, and MLTCs (having two or more chronic diseases). Among these variables, age category, sex, and presence of disabilities using the ADL and Barthel index were significantly different across MLTC groups. Tables 2, 3 show the demographics for all participants classified based on disabilities using the ADL and Barthel index, respectively.

**Table 1:** Demographics and clinical characteristics based on MLTCs.

Factors	No chronic disease *n* = 72	One chronic disease *n* = 92	MLTCs *n* = 160	p*
Age categories, n (% within MLTCs)				<0.001
< 65 years	53 (73.6)	57 (62.0)	71 (44.4)	
≥ 65 years	19 (26.4)	35 (38.0)	89 (55.6)	
Sex				0.28
Females, n (% within MLTCs)	48 (66.7)	50 (54.3)	95 (59.4)	
Males, n (% within MLTCs)	24 (33.3)	42 (45.7)	65 (40.6)	
BMI, categories				0.82
Normal	21 (29.2)	21 (22.8)	41 (25.6)	
Overweight	30 (41.7)	38 (41.3)	62 (38.8)	
Obese	21 (29.2)	33 (35.9)	57 (35.6)	
ADL disabilities				0.004
Yes	18 (25.0)	23 (25.0)	68 (42.5)	
No	54 (75.0)	69 (75.0)	92 (57.5)	
Barthel index for disabilities				0.007
Yes	27 (37.5)	41 (44.6)	93 (58.1)	
No	45 (62.5)	51 (55.4)	67 (41.9)	

MLTCs, Multiple long-term conditions; BMI, Body mass index; ADL, Activities of daily living. *p indicates the p-value that was based on the chi-square test.

**Table 2:** Demographics and clinical characteristics based on disabilities using ADL.

Factors	ADL disabilities No *n* = 215	ADL disabilities Yes *n* = 109	p*
Age categories, n (% within ADL)			0.010
< 65 years	131 (60.9)	50 (45.9)	
≥ 65 years	84 (39.1)	59 (54.1)	
Sex, n (% within ADL)			0.002
Females	115 (53.5)	78 (71.6)	
Males	100 (46.5)	31 (28.4)	
BMI, categories, n (% within ADL)			0.027
Normal	61 (28.4)	22 (20.2)	
Overweight	91 (42.3)	39 (35.8)	
Obese	63 (29.3)	48 (44.0)	

BMI, Body mass index; ADL, Activities of daily living. *p indicates the *p*-value that was based on the chi-square test.

**Table 3:** Demographics and clinical characteristics based on disabilities using the Barthel index.

Factors	Barthel index disabilities No *n* = 163	Barthel index disabilities Yes *n* = 161	p*
Age categories, n (% within Barthel)			0.075
< 65 years	98 (60.1)	83 (51.6)	
≥ 65 years	65 (39.9)	78 (54.5)	
Sex, n (% within Barthel)			0.026
Females	88 (54.0)	105 (65.2)	
Males	75 (46.0)	56 (34.8)	
BMI, categories, n (% within Barthel)			0.27
Normal	41 (25.2)	42 (26.1)	
Overweight	72 (44.2)	58 (36.0)	
Obese	50 (30.7)	61 (37.9)	

BMI, Body mass index. *p indicates the p-value that was based on the chi-square test.

The results of the multivariable binary logistic regression for the association between MLTCs as a risk factor and the presence of disabilities using ADL are shown in Table 4. MLTCs (two chronic diseases or more) were associated with the risk of having any kind of disabilities using ADL (OR 1.99, 95% confidence interval (95%CI) [1.04, 3.82], *p* = 0.037) after adjustments for age, sex, and BMI. There was no significant association between having one chronic disease and the risk of having any kind of disabilities using ADL (OR 0.98, 95% CI [0.47, 2.04], *p* = 0.95) after adjustments for age, sex, and BMI.

**Table 4:** Binary logistic regression for the association between MLTCs and disabilities using ADL.

*n* = 324	OR [95%CI]	*p*-value
MLTCs	1.99 [1.04, 3.82]	0.037
One chronic disease	0.98 [0.47, 2.04]	0.95
No chronic disease	Ref	Ref

MLTCs, Multiple long-term conditions; OR, odds ratio; CI: confidence interval. The model was adjusted for age, sex, and BMI.

The results of the multivariable binary logistic regression for the association between MLTCs as a risk factor and the presence of disabilities using the Barthel index are shown in Table 5. MLTCs (two chronic diseases or more) were associated with the risk of having any kind of disabilities using the Barthel index (OR 2.27, 95%CI [1.26, 4.11], *p* = 0.007) after adjustments for age, sex, and BMI. The results indicate that community-dwelling adults aged 50 years and older were 2.27 times more likely to have any kind of disabilities using the Barthel index in this population. There was no significant association between having one chronic disease and the risk of having any kind of disabilities using the Barthel index (OR 1.40, 95%CI [0.73, 2.66], *p* = 0.31), after adjustments for age, sex, and BMI.

**Table 5:** Binary logistic regression for the association between MLTCs and disabilities using the Barthel index.

*n* = 324	OR [95%CI]	*p*-value
MLTCs	2.27 [1.26, 4.11]	0.007
One chronic disease	1.40 [0.73, 2.66]	0.31
No chronic disease	Ref	Ref

MLTCs, multiple long-term conditions; OR, odds ratio; CI, confidence interval. The model was adjusted for age, sex, and BMI.

## Discussion

The present study was the first that examined the prevalence of MLTCs and disabilities among adults aged 50 years and older in Saudi Arabia. The findings of this study showed that MLTCs were significantly and strongly associated with disabilities in this population using the ADL and Barthel index.

This study added to the literature regarding the prevalence of MLTCs and disabilities in Saudi Arabia using more than one scale for disabilities (i.e., ADL and Barthel index). Approximately half of the participants had MLTCs, and approximately the third to half had any kind of disabilities using either Arabic versions of the ADL or the Barthel index. The prevalence of disabilities in our sample was consistent with previous studies conducted among the older adult in Poland and Spain (21, 37). In Poland, the prevalence of disabilities measured by ADL was 29% of a sample composed of 100 adults aged >65 years old (21). In Spain, a large study conducted among 4,995 older adults (>65 years old) demonstrated that 51.25% of participants have a disabilities according to the Barthel index (37).

The present study showed that participants aged 50 years and older with MLTCs were associated with a disabilities using ADL and the Barthel index. Similar results were obtained in the studies carried out by Jędrzejczyk et al. (21), and Forjaz et al. (37), and Calderón-Larrañaga et al. (38). In the research study of Jędrzejczyk et al., it was proved that an increase in the number of comorbidities contributes to a reduction in the level of performing complex ADL among older adult aged >65 years old (21). A study by Calderon-Larranaga et al. suggested that the speed at which diseases accumulate over time affects the development of disabilities among the older adult aged >60 years (38). Specific patterns have been noted in osteoarticular, diabetes, and mental health disorders that are particularly strongly associated with functional disabilities and lower quality of life among older adult aged ≥65 years old (37).

The association between chronic conditions and disabilities is bidirectional. Individuals with disabilities are more likely to develop chronic conditions, and those with chronic conditions are more likely to develop disabilities (34, 37, 39–41). However, it is important to note that not everyone with a chronic condition will develop a disabilities, and not everyone with a disabilities will develop a chronic condition. A study using data from the National Health Interview Survey, which included adults with lifelong disabilities (*n* = 2,619) and adults without limitations (*n* = 122,395), found that those with lifelong disabilities (including physical, mental, intellectual/developmental, and sensory disabilities) were more likely to have chronic conditions than those without limitations. This suggests that having a disabilities may increase the risk of developing poor health (40). More recent evidence identified a link between disabilities status and the likelihood of being diagnosed with chronic diseases (41). Their results showed that older adults with significant difficulties related to disabilities had a higher prevalence (57.0%) of self-reported chronic disease diagnoses (41).

However, the differences between our study sample compared to the previous literature should be mentioned. The study participants were 50 years and older, while previous studies included the older adult aged ≥65 years old (21, 37). The justification for including participants aged 50 years old is due to the high prevalence of chronic diseases (i.e., hypertension, diabetes mellitus, osteoarthritis, and low back pain) among adults in the Saudi population (22, 42, 43). The latest national prevalence of hypertension in the Saudi population was carried out by Alenazi and Alqahtani and showed that the prevalence of hypertension increased at the age of 40 years and peaked at the age of 65 years and older (28). In addition, osteoarthritis has been reported in a systematic review and meta-analysis with a total of 24,625 participants and estimated an overall prevalence of 16.13% of OA among people living in the Gulf Cooperation Council countries (22).

Existing studies that have examined the mechanisms of how MLTCs progress to disabilities are scarce and have focused mainly on physical pathways. For instance, studies have demonstrated that inflammation plays an important role in the link between MLTCs and functioning limitations (44, 45). From a biological perspective, it is plausible that the rapid development of MLTCs may be a consequence of an increased degree of molecular damage, which reduces physiological reserve and thus increases the risk for transition from disease to disabilities. Moreover, the loss of homeostatic capacity linked with the faster rate of disease accumulation might lead to reduced physical and cognitive function and thereby lead to a disabilities (46). Therefore, attention and prevention of MLTCs can be one of the most appropriate factors to prevent disabilities in older adults.

The clinical implications and health policy may benefit from the current study findings (47, 48). The high prevalence of MLTCs and disabilities in community-dwelling adults aged 50 years and older requires the development of targeted interventions to improve their functional outcomes. By identifying the specific factors that contribute to the development and progression of MLTCs and disabilities, healthcare providers can develop personalized treatment approaches that address the unique needs of each patient. The prioritization of healthcare resources based on the identified etiological factors can help to optimize the allocation of resources and improve the efficiency of care delivery. Furthermore, the development of preventive and therapeutic strategies for MLTCs can help in reducing the burden of disabilities. Therefore, this study highlights the importance of continued research in this area and the need for the development of evidence-based interventions to address the complex needs of this population.

Nevertheless, the following limitations should be considered. First, the information about chronic conditions has been collected through self-reported and memory-related diseases, which might bias the findings. Recall bias may include difficulty recalling or accurately reporting past events or experiences, leading to under-or over-estimate symptoms or functional limitations. Additionally, older adults with cognitive impairments such as dementia or Alzheimer’s disease may affect the ability to accurately complete self-report questionnaires or participate in testing procedures, leading to incomplete or inaccurate data. Self-reported measures may be influenced by social desirability bias, where participants may respond in a manner that they believe is socially acceptable, leading to over-or under-estimation of symptoms, diseases, or physical functions. Second, the information about chronic diseases was limited due to the lack of inclusion of some chronic conditions, such as low back pain and mental illness. Therefore, the prevalence of the MLTCs might be underestimated. The current sample was convenient (a non-random sampling), and this might affect the results of this study. Convenient sampling selected participants based on their availability rather than random selection. This may lead to a selection bias resulting in over-representation or under-representation of groups in the sample. Convenience sampling may result in a lack of diversity, leading to limited generalizability of the findings to other populations. Although the sample size was calculated, the current sample was relatively small, which prevented us from performing subgroup analysis based on age groups and sex. However, we included age and sex as covariates in the analysis to control for their influence on the results. Future research should examine sex-specific disabilities and MLTCs among community-dwelling adults. Disabilities was limited to only two measures that were self-reported. Therefore, future studies should examine disabilities using subjective and objective measures. This study was observational using a cross-sectional design. Further research is needed to examine whether MLTCs are a potential disabilities incidence in this population using prospective longitudinal studies.

## Conclusion

Approximately half of the participants had MLTCs. Approximately a third to half of the participants had any type of disabilities, depending on the disabilities scale. This study found that MLTCs were significantly associated with any kind of disabilities in community-dwelling Saudi adults living in Riyadh according to the ADL and Barthel index. Further studies are needed to establish the causal relationship between comorbidities and disabilities development using a prospective design with rigorous tools for examining MLTCs and disabilities. Comprehensive geriatric care is required to prevent MLTCs and decrease the risk of functional deterioration and disabilities.

## Data Availability

The raw data supporting the conclusions of this article will be made available by the authors, without undue reservation.
